# Supplementary material for: Poor outcome of pediatric patients with acute myeloid leukemia harboring high FLT3/ITD allelic ratios
Source: Nat Commun. 2022 Jun 27;13:3679. doi: 10.1038/s41467-022-31489-9 (PMC9237020; doi:10.1038/s41467-022-31489-9)
Supplement: Supplementary file 1 — Supplementary Information [file 41467_2022_31489_MOESM1_ESM.pdf]

Supplement Tables

Supplementary Table 1 AAML03P1 Therapeutic Regimen

Course and drugs	Does	Days
Induction Course Ⅰ		
Cytarabine	100 mg/m^2/dose twice per day IV	1-10
Daunomycin	50 mg/m^2/dose IV	1, 3, 5
Etoposide	100 mg/m^2/dose IV	1-5
Gemtuzumab Ozogamicin	3 mg/m^2/dose IV over 2 hours	6
Induction Course Ⅱ		
Cytarabine	100 mg/m^2/dose twice per day IV	1-8
Daunomycin	50 mg/m^2/dose IV	1, 3, 5
Etoposide	100 mg/m^2/dose IV	1-5
Intensification course Ⅰ		
Cytarabine	1,000 mg/m^2/dose twice per day IV	1-5
Etoposide	150 mg/m^2/dose IV	1-5
Intensification course Ⅱ		
Mitoxantrone	12mg/m^2/dose IV	3-6
Cytarabine	1,000 mg/m^2/dose twice per day IV	1-4
Gemtuzumab	3 mg/m^2/dose IV over 2 hours	7
Intensification course Ⅲ		
Cytarabine	3,000 mg/m^2/dose twice per day IV	1, 2, 8, 9
Escherichia coli L-asparaginase	6,000 U/m^2/dose IM	2, 9

Abbreviations: IM, intramuscular; IV, intravenous.

Supplementary Table 2 AAML0531 Therapeutic Regimen

Course and drugs	Does	Days
Induction Course Ⅰ		
Cytarabine	100 mg/m^2/dose twice per day IV	1-10
Daunomycin	50 mg/m^2/dose IV	1, 3, 5
Etoposide	100 mg/m^2/dose IV	1-5
Gemtuzumab Ozogamicin	3 mg/m^2/dose IV over 2 hours	6
Induction Course Ⅱ		
Cytarabine	100 mg/m^2/dose twice per day IV	1-8
Daunomycin	50 mg/m^2/dose IV	1, 3, 5
Etoposide	100 mg/m^2/dose IV	1-5
Intensification course Ⅰ		
Cytarabine	1,000 mg/m^2/dose twice per day IV	1-5
Etoposide	150 mg/m^2/dose IV	1-5
Intensification course Ⅱ		
Mitoxantrone	12mg/m^2/dose IV	3-6
Cytarabine	1,000 mg/m^2/dose twice per day IV	1-4
Gemtuzumab	3 mg/m^2/dose IV over 2 hours	7
Intensification course Ⅲ		
Cytarabine	3,000 mg/m^2/dose twice per day IV	1, 2, 8, 9
Escherichia coli L-asparaginase	6,000 U/m^2/dose IM	2, 9

Abbreviations: IM, intramuscular; IV, intravenous.

Supplementary Table 3 AAML1031 Therapeutic Regimen

Course and drugs	Does	Days
Induction Course Ⅰ		
Cytarabine	100 mg/m^2/dose twice per day IV	1-10
Daunomycin	50 mg/m^2/dose IV	1, 3, 5
Etoposide	100 mg/m^2/dose IV	1-5
Induction Course Ⅱ		
Cytarabine	1000 mg/m^2/dose twice per day IV	1-4
Mitoxantrone	12mg/m^2/dose IV	3-6
Intensification course Ⅰ		
Cytarabine	1,000 mg/m^2/dose twice per day IV	1-5
Etoposide	150 mg/m^2/dose twice per day IV	1-5
Intensification course Ⅱ		
Cytarabine	3,000 mg/m^2/dose twice per day IV	1,2,8,9
Escherichia coli L-asparaginase	6,000 mg/m^2/dose IM	2, 9

Abbreviations: IM, intramuscular; IV, intravenous.

Supplementary Table 4 CCG-2961 Therapeutic Regimen

Course and drugs	Does	Days
Induction Course Ⅰ		
Idarubicin	5 mg/m^2/dose IV over half an hour per day	0-3
Cytarabine	200 mg/m^2/dose IV per day	0-3
Etoposide	100 mg/m^2/dose IV per day	0-3
Thioguanine	50 mg/m^2/dose oral twice per day	0-3
Dexamethasone	2 mg/m^2/dose three times per day	0-3
Induction Course Ⅱ		
Cytarabine	200 mg/m^2/dose IV per day	10-13
Etoposide	100 mg/m^2/dose IV per day	10-13
Thioguanine	50 mg/m^2/dose oral twice per day	10-13
Dexamethasone	2 mg/m^2/dose three times per day	10-13
Daunorubicin	20 mg/m^2/dose per day	10-13
Filgrastim		Beginning at Day 16
Consolidation course Ⅰ		
Fludarabine monophosphate,	10.5 mg/m^2 loading dose→30.5 mg/m^2 per day	0-1
Cytarabine	390 mg/m^2 loading dose→2400 mg/m^2 per day	2-4
Idarubicin	12 mg/m^2/dose IV over half an hour per day	0-2
Filgrastim		Beginning at Day 6
Intensification course Ⅰ		
Cytarabine	3 g/m^2/dose IV	0,1,7,8
Escherichia coli L-asparaginase	6000 U/m^2/dose IM	1,8

Abbreviations: IM, intramuscular; IV, intravenous.

Supplementary Table 5 Baseline Characteristics of FLT3/ITD positive pediatric AML in the GO and No-GO cohorts.

Characteristics	No-GO(n=80)	GO (n=89)	*P* value
Gender, n(%)			0.492
Male	50 (62.5%)	51 (57.3%)	
Female	30 (37.5%)	38 (42.7%)	
Age(y), median(range)	12.1 (3.0-17.9)	10.6 (1.8-17.9)	0.012
FAB Category			0.042
M0	1 (1.7%)	1 (1.4%)	
M1	21 (35.0%)	13 (18.1%)	
M2	11 (18.3%)	22 (30.6%)	
M4	16 (26.7%)	18 (25.0%)	
M5	8 (13.3%)	18 (25.0%)	
M6	3 (5.0%)	0 (0.0%)	
Chemotherapy protocol, n(%)			0.003
AAML03P1	3 (3.8%)	16 (18.0%)	
AAML0531	77 (96.2%)	73 (82.0%)	
Initial WBC($\times 10^9$/L),median(range)	68.2 (0.2-473.1)	68.3 (3.1-447.3)	0.952
PB blast（%）	60.5 (0.0-98.0)	65.0 (4.0-98.0)	0.361
BM blast (%)	80.0 (20.0-98.0)	80.0 (25.0-99.0)	0.879
Karyotype			0.196
Normal	44 (57.1%)	41 (48.8%)	
inv(16)	4 (5.2%)	2 (2.4%)	
MLL	5 (6.5%)	2 (2.4%)	
t(8;21)	1 (1.3%)	4 (4.8%)	
Other	23 (29.9%)	35 (41.7%)	
Risk group, n(%)			0.171
Low risk	11 (14.1%)	10 (11.6%)	
Standard risk	19 (24.4%)	12 (14.0%)	
High risk	48 (61.5%)	64 (74.4%)	
CNSL, n(%)			0.448
Yes	78 (97.5%)	84 (94.4%)	
No	2 (2.5%)	5 (5.6%)	
CEBPA status, n(%)			0.738
CEBPA wild-type	74 (93.7%)	83 (95.4%)	
CEBPA mutation	5 (6.3%)	4 (4.6%)	
NPM1 status, n(%)			0.348
NPM1 wild-type	64 (81.0%)	76 (86.4%)	
NPM1 mutation	15 (19.0%)	12 (13.6%)	

Supplementary Table 5 (continue)

Characteristics	No-GO(n=80)	GO (n=89)	*P* value
WT1 status, n(%)			0.589
WT1 wild-type	62 (78.5%)	72 (81.8%)	
WT1 mutation	17 (21.5%)	16 (18.2%)	
CR status at end of course 1			0.898
CR	50 (63.3%)	58 (65.9%)	
Not in CR	28 (35.4%)	28 (31.8%)	
Death	1 (1.3%)	2 (2.3%)	
CR status at end of course 2			0.396
CR	58 (73.4%)	66 (80.5%)	
Not in CR	20 (25.3%)	14 (17.1%)	
Death	1 (1.3%)	2 (2.4%)	
SCT in 1st CR			0.425
No	37 (56.1%)	47 (62.7%)	
Yes	29 (43.9%)	28 (37.3%)	
FLT3/ITD allelic ratio			0.839
$<$0.5	39 (48.8%)	42 (47.2%)	
≥0.5	41 (51.2%)	47 (52.8%)	

Abbreviation: WBC, white blood cell counts; PB blast, peripheral blood blast; BM, bone marrow blast; CNSL, central nervous system leukemia; CEBPA,CCAAT/enhancer binding protein alpha; FLT3-ITD, fms-related tyrosine kinase 3; NPM1,nucleophosmin 1; WT1,wilms tumor 1; CR,complete remission; SCT, stem cell transplantation.;GO, Gemtuzumab ozogamicin treatment.The qualitative data were analyzed using the chi-square test and the quantitative data were compared using the Student's t-test (two-tailed).

Supplementary Table 6 Baseline Characteristics of FLT3/ITD≥0.5 pediatric AML in the GO and No-GO cohorts.

Characteristics	No-GO(n=41)	GO (n=47)	*P* value
Gender, n(%)			0.918
Male	24 (58.5%)	27 (57.4%)	
Female	17 (41.5%)	20 (42.6%)	
Age(y), median(range)	13.1 (3.0-17.9)	10.5 (2.9-17.9)	0.122
Initial WBC($\times 10^9$/L),median(range)	71.9 (0.9-446.0)	70.6 (3.1-447.3)	0.751
PB blast（%）	72.0 (0.0-97.0)	72.0 (0.0-97.0)	0.806
BM blast (%)	85.8 (40.6-98.0)	87.0 (30.0-99.0)	0.560
Karyotype			0.283
Normal	19 (48.7%)	25 (55.6%)	
inv(16)	1 (2.6%)	1 (2.2%)	
MLL	4 (10.3%)	0 (0.0%)	
t(8;21)	1 (2.6%)	2 (4.4%)	
Other	14 (35.9%)	17 (37.8%)	
CNSL, n(%)			0.640
Yes	40 (97.6%)	45 (95.7%)	
No	1 (2.4%)	2 (4.3%)	
CR status at end of course 1			0.696
CR	26 (65.0%)	26 (56.5%)	
Not in CR	13 (32.5%)	18 (39.1%)	
Death	1 (2.5%)	2 (4.3%)	
CR status at end of course 2			0.854
CR	27 (67.5%)	31 (68.9%)	
Not in CR	12 (30.0%)	12 (26.7%)	
Death	1 (2.5%)	2 (4.4%)	
SCT in 1st CR			0.481
No	17 (51.5%)	21 (60.0%)	
Yes	16 (48.5%)	14 (40.0%)	

Abbreviation: WBC, white blood cell counts; PB blast, peripheral blood blast; BM, bone marrow blast; CNSL, central nervous system leukemia; CEBPA,CCAAT/enhancer binding protein alpha; FLT3-ITD, fms-related tyrosine kinase 3; NPM1,nucleophosmin 1; WT1,wilms tumor 1; CR,complete remission; SCT, stem cell transplantation.;GO, Gemtuzumab ozogamicin treatment.The qualitative data were analyzed using the chi-square test and the quantitative data were compared using the Student's t-test (two-tailed).
