# Supplementary material for: Poor outcome of pediatric patients with acute myeloid leukemia harboring high FLT3/ITD allelic ratios
Source: Nat Commun. 2022 Jun 27;13:3679. doi: 10.1038/s41467-022-31489-9 (PMC9237020; doi:10.1038/s41467-022-31489-9)
Supplement: Supplementary file 3 — Reporting Summary [file 41467_2022_31489_MOESM3_ESM.pdf]

nature portfolio

Corresponding author(s): Dun-hua Zhou

Last updated by author(s): May 20, 2022

Reporting Summary

Nature Portfolio wishes to improve the reproducibility of the work that we publish. This form provides structure for consistency and transparency in reporting. For further information on Nature Portfolio policies, see our Editorial Policies and the Editorial Policy Checklist.

Statistics

For all statistical analyses, confirm that the following items are present in the figure legend, table legend, main text, or Methods section.

n/a	Confirmed	
☐	☒	The exact sample size (*n*) for each experimental group/condition, given as a discrete number and unit of measurement
☒	☐	A statement on whether measurements were taken from distinct samples or whether the same sample was measured repeatedly
☐	☒	The statistical test(s) used AND whether they are one- or two-sided<br>*Only common tests should be described solely by name; describe more complex techniques in the Methods section.*
☐	☒	A description of all covariates tested
☐	☒	A description of any assumptions or corrections, such as tests of normality and adjustment for multiple comparisons
☐	☒	A full description of the statistical parameters including central tendency (e.g. means) or other basic estimates (e.g. regression coefficient) AND variation (e.g. standard deviation) or associated estimates of uncertainty (e.g. confidence intervals)
☐	☒	For null hypothesis testing, the test statistic (e.g. *F*, *t*, *r*) with confidence intervals, effect sizes, degrees of freedom and *P* value noted<br>*Give P values as exact values whenever suitable.*
☐	☒	For Bayesian analysis, information on the choice of priors and Markov chain Monte Carlo settings
☐	☒	For hierarchical and complex designs, identification of the appropriate level for tests and full reporting of outcomes
☒	☐	Estimates of effect sizes (e.g. Cohen's *d*, Pearson's *r*), indicating how they were calculated

Our web collection on statistics for biologists contains articles on many of the points above.

Software and code

Policy information about availability of computer code

Data collection	ALL data are generated by the Therapeutically Applicable Research to Generate Effective Treatments (https://ocg.cancer.gov/programs/target) and it is not necessary for us to collect the data by software.
Data analysis	All statistical analysis by SPSS statistical software version 22.0 and EmpowerStats statistical software version 2.2(http://www.empowerstats.cn/).

For manuscripts utilizing custom algorithms or software that are central to the research but not yet described in published literature, software must be made available to editors and reviewers. We strongly encourage code deposition in a community repository (e.g. GitHub). See the Nature Portfolio guidelines for submitting code & software for further information.

Data

Policy information about availability of data

All manuscripts must include a data availability statement. This statement should provide the following information, where applicable:

- Accession codes, unique identifiers, or web links for publicly available datasets
- A description of any restrictions on data availability
- For clinical datasets or third party data, please ensure that the statement adheres to our policy

The data sets used and/or analyzed during the current study are available from the TARGET dataset (https://ocg.cancer.gov/programs/target) or Figshare database

(DOI:10.6084/m9.figshare.19809565). The remaining data are available in the Article and Supplementary Information. Source data are provided with this paper. Source data is included with this Article.

Human research participants

Policy information about studies involving human research participants and Sex and Gender in Research.

Reporting on sex and gender	Among the TARGET group, 974 (52.5%) were male and 883 (47.5%) were female, and the median age in our cohort was 9.5 years old. The clinical characteristics between them are not statistically significant.
Population characteristics	In total, 1,857 pediatric AML patients were enrolled in this study. Among the TARGET group, 974 (52.5%) were male and 883 (47.5%) were female, and the median age in our cohort was 9.5 years old. The demographic, laboratory, and clinical characteristics of pediatric AML patients were compared based on the FLT3/ITD status (Table 1). The prevalence of FLT3/ITD mutation in the whole cohort was 18.4%. Mutant FLT3/ITD was more common in males compared with wild-type in terms of gender distribution (57.8% vs. 51.3%, P=0.029). The median age of mutant FLT3/ITD was higher than that of wild-type (11.9 years vs. 8.4 years, P<0.001) and mutant FLT3/ITD was far more common in older patients (>10 years) (66.6% of patients harbored mutant FLT3/ITD, compared with 43.5% of patients with wild-type). The initial median WBC of mutant FLT3/ITD patients was higher than that of wild-type patients (70.6×109/L vs. 25.7×109/L, P=0.004), and was more common in patients with WBC>50×109/L (60.1% vs. 34.7%, P=0.011). Both peripheral blood (PB) blasts and bone marrow (BM) blasts among children harboring FLT3/ITD mutation were significantly higher than those harboring wild-type (PB blast: 65% vs. 41%, P<0.001; BM blast: 80% vs. 68%, P<0.001).
Recruitment	We download demographic, clinical characteristics and laboratory data of AML in children under the age of 18 from the TARGET dataset(April 28, 2021). From September 1996 to December 2016, 2,064 pediatric AML (non-M3) cases were enrolled in the TARGET database: 121 were exclude (secondary AML myeloid, n=35; Down syndrome, n=86), 86 were unable to evaluate (loss of FLT3/ITD information, n=4; insufficient data on therapy, n=82), and finally, 1,857 childhood AML were enrolled in our research.
Ethics oversight	The study was approved by the Ethics Committees of Office of Cancer Genomics

Note that full information on the approval of the study protocol must also be provided in the manuscript.

Field-specific reporting

Please select the one below that is the best fit for your research. If you are not sure, read the appropriate sections before making your selection.

☒ Life sciences ☐ Behavioural & social sciences ☐ Ecological, evolutionary & environmental sciences

For a reference copy of the document with all sections, see nature.com/documents/nr-reporting-summary-flat.pdf

Life sciences study design

All studies must disclose on these points even when the disclosure is negative.

Sample size	The TARGET program was launched in the United States to provide data and follow-up on various aspects of pediatric oncology. The program included four pediatric acute myeloid leukemia clinical trials, and children under 18 years of age and with acute myeloid leukemia were eligible to participate in TARGET after providing informed consent. Similar sample size calculations were performed by previous clinical trials in the United States and included 2064 cases of pediatric acute myeloid leukemia. Here, we included data from September 1996 to December 2016, with the final inclusion of the present study population comprising 1835 cases.
Data exclusions	From September 1996 to December 2016, 2,064 pediatric AML (non-M3) cases were enrolled in the TARGET database: 121 were exclude (secondary AML myeloid, n=35; Down syndrome, n=86), 86 were unable to evaluate (loss of FLT3/ITD information, n=4; insufficient data on therapy, n=82), and finally, 1,857 childhood AML were enrolled in our research.
Replication	There are two main steps to the analyses reported in the study, and we developed two separate models which were both replicated:<br>Step 1) We compare the baseline characteristics of study participants by FLT3/ITD status classification by single factor analysis and we further compare the baseline characteristics of high FLT3/ITD high AR and low AR and this is replicated.<br>Step 2) We developed a model to assess the optimal threshold of FLT3/ITD AR. Through this model, the threshold can be calculated repeatedly by different data.
Randomization	Our study is a retrospective study, and no randomization has been performed.
Blinding	Blinding was not appicable in our study, as it is not an intervention study.

Reporting for specific materials, systems and methods

We require information from authors about some types of materials, experimental systems and methods used in many studies. Here, indicate whether each material, system or method listed is relevant to your study. If you are not sure if a list item applies to your research, read the appropriate section before selecting a response.

Materials & experimental systems

n/a	Involved in the study
☒	☐ Antibodies
☒	☐ Eukaryotic cell lines
☒	☐ Palaeontology and archaeology
☒	☐ Animals and other organisms
☐	☒ Clinical data
☒	☐ Dual use research of concern

Methods

n/a	Involved in the study
☒	☐ ChIP-seq
☒	☐ Flow cytometry
☒	☐ MRI-based neuroimaging

Clinical data

Policy information about clinical studies

All manuscripts should comply with the ICMJE guidelines for publication of clinical research and a completed CONSORT checklist must be included with all submissions.

Clinical trial registration	Clinical trial registration number include (NCT00070174), (NCT00372593), (NCT01371981), and (NCT00002798).
Study protocol	All trials protocol included in project TARGET are available via clinicaltrials.gov by searching trial registration number include (NCT00070174), (NCT00372593), (NCT01371981), and (NCT00002798).
Data collection	We download demographic, clinical characteristics and laboratory data of AML in children under the age of 18 from the TARGET dataset(April 28, 2021). From September 1996 to December 2016, 2,064 pediatric AML (non-M3) cases were enrolled in the TARGET database: 121 were exclude (secondary AML myeloid, n=35; Down syndrome, n=86), 86 were unable to evaluate (loss of FLT3/ITD information, n=4; insufficient data on therapy, n=82), and finally, 1,857 childhood AML were enrolled in our research.
Outcomes	The primary outcome is to identify the cut-off value on FLT3/ITD AR and the second outcome is to evaluate different treatment strategy for FLT3/ITD-positive AML pediatric patients. The primary outcome are assessed by the restrictive cubic spline function and the second outcome are assessed by survival analysis.
